# Preparation, Marriage Chemistry and Applications of Graphene Quantum Dots–Nanocellulose Composite: A Brief Review

**DOI:** 10.3390/molecules26206158

**Published:** 2021-10-12

**Authors:** Wan Hazman Danial, Nur Fathanah Md Bahri, Zaiton Abdul Majid

**Affiliations:** 1 Department of Chemistry, Kulliyyah of Science, International Islamic University Malaysia, Kuantan 25200, Pahang, Malaysia; fathanahbahri93@gmail.com; 2 Department of Chemistry, Faculty of Science, Universiti Teknologi Malaysia, Johor Bahru 81310, Johor, Malaysia; zaitonmajid@utm.my

**Keywords:** graphene quantum dots, nanocellulose, composite

## Abstract

Graphene quantum dots (GQDs) are zero-dimensional carbon-based materials, while nanocellulose is a nanomaterial that can be derived from naturally occurring cellulose polymers or renewable biomass resources. The unique geometrical, biocompatible and biodegradable properties of both these remarkable nanomaterials have caught the attention of the scientific community in terms of fundamental research aimed at advancing technology. This study reviews the preparation, marriage chemistry and applications of GQDs–nanocellulose composites. The preparation of these composites can be achieved via rapid and simple solution mixing containing known concentration of nanomaterial with a pre-defined composition ratio in a neutral pH medium. They can also be incorporated into other matrices or drop-casted onto substrates, depending on the intended application. Additionally, combining GQDs and nanocellulose has proven to impart new hybrid nanomaterials with excellent performance as well as surface functionality and, therefore, a plethora of applications. Potential applications for GQDs–nanocellulose composites include sensing or, for analytical purposes, injectable 3D printing materials, supercapacitors and light-emitting diodes. This review unlocks windows of research opportunities for GQDs–nanocellulose composites and pave the way for the synthesis and application of more innovative hybrid nanomaterials.

## 1. Introduction

Over the past few years, many researchers have developed an interest in investigating carbon-based nanomaterials, such as graphene and graphene quantum dots (GQDs) [1,2,3,4]. Graphene is one layer of sp^2^-hybridised carbon atoms arranged in a honeycomb lattice [5], whereas GQDs are chopped fragments of a few graphene sheets (<10 nm lateral dimension) and <10 graphene layers which form final particles [6]. GQDs are used in various applications owing to their higher photostability, low cytotoxicity, strong photoluminescence, dispersibility in water, fluorescence and excellent biocompatibility [7,8]. Furthermore, the characteristics of GQDs vary according to morphology, size, doping concentration and type [9]. Owing to these properties and the ability to fine-tune them, GQDs are investigated for different applications in biomedicine [10], catalyst development [11], energy [12] and sensing and photo electronics [6]. Different bottom-up and top-down strategies have been used to produce GQDs, such as organic synthesis [13], hydrothermal [14], microwave irradiation [15], chemical exfoliation [16] and electrochemical exfoliation [3,17].

Furthermore, many researchers have also conducted extensive carbohydrate polymer research using cellulose-based nanomaterials. Cellulose is an abundantly available, naturally occurring organic polymer that is composed of repeating units of *β*-glucopyranose rings that are covalently linked to one another with a *β* 1-4 glycosidic bond [18,19]. Cellulose nanowhiskers, or nanocellulose, are cellulose particles present in the form of crystals or fibres [20]. These particles are few micrometres in length and have a diameter <100 nm. These fibres are lightweight, biodegradable and also have a higher water-binding capacity [21]. Cellulosic nanomaterials display a larger specific surface area; therefore, they can form many hydrogen bonds. This hydrogen bond-forming capability helps the material develop a dense and strong network [22]. Many different kinds of nano-cellulosic materials are described in the literature, such as cellulose nanocrystals (CNCs) [23], nanofibrillated cellulose (NFCs) [24] and bacterial nanocellulose (BNCs) [25]. As nanocellulose can be derived from different cellulosic sources, each displays varying properties and some are derived from natural sources. For instance, the properties of the bacterial nanocelluloses are based on different bacterial sources, whereas the properties of the cellulose nanocrystals and nanofibrillated celluloses are based on sources such as tunicin or plants [26]. Nanocellulose has garnered significant research interest as a promising nanomaterial that can revolutionise multiple fields, such as the pharmaceutical field [27], engineering [28], electronics [29] and health and environmental protection [30].

GQD-nanocellulose is described as a hybrid material that contains nanocellulose and GQDs, which synergistically improves the properties of every individual component, such as their stability and mechanical strength [31]. The addition of GQDs to a nanocomposite material can improve its final tensile strength, stiffness and the toughness of these GQDs–nanocellulose structures, irrespective of their GQD oxidation type and nanocellulose orientation [32]. These structures are designed to exhibit higher conductivity, cycle stability and higher specific capacitance [32]. Therefore, GQDs–nanocellulose composites are designed for use in many applications, such as fluorescence films, bendable and portable paper electronics, and hydrogels. The numerous -COOH and -OH functional groups are present on the surfaces of GQDs result in the formation of hydrogen bonds at the GQDs–nanocellulose interface. This can significantly affect the GQDs–nanocellulose supercells, nanocellulose lattice parameters and the morphological properties of nanocellulose, such as the dihedral angle differences in the hydroxymethyl groups, axial tilt of molecular chains and the flipping motion of terminal groups [33]. In addition, the composites made from these renewable nanomaterials offer a greener approach than petroleum-derived composites and exhibit great potential for various technological applications [34]. Moreover, this nanomaterial combination has recently emerged as a new class of hybrid material due to its exceptional features and notable synergistic effects [35].

In this review, the preparation, marriage chemistry and applications of GQDs–nanocellulose composites are discussed. Although these composites have been investigated in recent years, reviews that focus solely on GQDs–nanocellulose composites have not yet been reported. This review also provides some insight into the development of the fluorescent hydrogel functions of the composites.

### 1.1. Graphene Quantum Dots (GQDs)

GQDs hold sp^2^ hybridised carbon single-layer nanocrystals and they are highly fluorescent, regardless of whether they are in an aqueous or solid state [36,37]. GQDs are a distinct from the type of carbon in carbon nanodots (CNDs) and polymer dots (PDs). This is because all carbon dots possess modified chemical groups, such as oxygen groups, on the surface [38]. However, each of them is of a different size and possesses different properties with which to perform their action. GQDs have an average lattice parameter of 0.24 nm, which corresponds to 100 in plane graphene lattice parameters [39]. On the contrary, CNDs are divided into carbon nanoparticles without a crystal lattice and have a spherical shape [40], while the grafted and cross-linked polymer chains of linear non-conjugated polymers form PDs [36].

GQDs are the simplest carbon dots with connected chemical groups on the surfaces or edges [41]. The surfaces or edges of GQDs contain triple carbene at the zigzag edges and oxygen groups at the graphene core. Additionally, the type of GQD edge plays a significant role in determining the material’s optical, electronic and magnetic properties [36]. GQDs are produced using either the top-down or bottom-up method. Both approaches use different parameters to produce GQDs [36]. The top-down method involves direct cutting of graphite or graphene-based materials via acid exfoliation [16], sonochemistry [42], solvothermal synthesis [43], electrochemistry [44,45,46,47], or chemical oxidation [48,49,50]. The advantages of these methods are an abundance of inexpensive precursor (graphite), the high graphitic nature and the formation of GQDs with high oxygen-containing functional groups, which renders good solubility and functionality. However, the drawbacks of these methods include harsh a reaction procedure, as well as the non-uniform size and thickness of the final product [51]. Examples of the bottom-up method of GQD production from molecular precursors include cyclodehydrogenation [52], pyrolysis [53] and solution chemistry [13]. Despite the difficult synthesis processes, the bottom-up approaches provide better control over the size and shape of the GQDs.

Graphene is widely used in many applications, such as electronics, solar cells and Li-ion batteries [54], whereas GQDs have attracted tremendous interest in photoluminescence [55], cell-imaging [56] and drug delivery [57,58], among many things. GQDs have a great advantage, as their properties can be adjusted by changing their band via doping to produce amine-functionalised GQDs [59], nitrogen-doped GQDs (N-GQDs) [60] and sulphur–nitrogen co-doped GQDs (S, N-GQDs) [61]. Furthermore, the photoluminescence colour of GQDs can also be changed from violet to yellow by setting the reactant concentration and temperature during the hydrothermal method [54].

### 1.2. Nanocellulose

**Figure 1:**
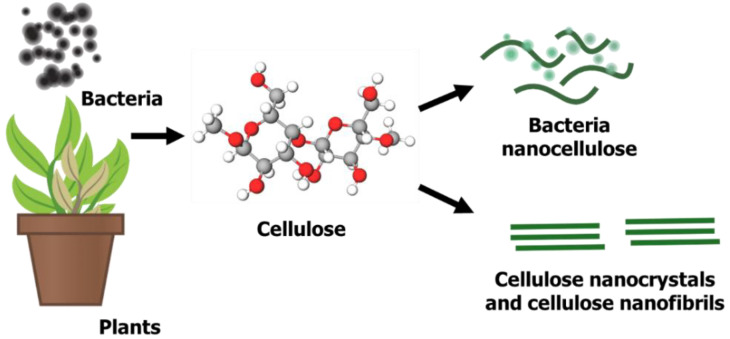
Formation of nanocellulose from cellulose-containing materials.

Nanocellulose, a sustainable and renewable nano-structured cellulose, has gained tremendous attention for its potential use in many applications due to its excellent surface chemistry, physical properties and remarkable biological properties [62,63]. Nanocellulose was first prepared in 1947 using sulfuric acid and hydrochloric acid hydrolysis from wood fibres and cotton fibres [64]. As illustrated in Figure 1, there are three types of nanocellulose from different precursors, bacteria nanocellulose, cellulose nanocrystals (CNCs) and cellulose nanofibrils (CNF) [65,66], which can be obtained from cellulose-containing precursors such as plants or bacteria. Plant cellulose is situated within a plant’s fibre walls, whereas bacteria produce exopolysaccharides to form microbial cellulose [67]. These bacterial isolates can normally be obtained from rotten vegetables and fruits. Similar to plant-derived cellulose, some parameters or conditions, such as carbon source, nitrogen source, temperature, pH and agitation, are measured to produce a high yield of bacterial cellulose [68,69]. Bacterial nanocellulose is pure in nature and has a low cytotoxicity, high pore distribution and high hydrophilicity due to the presence of OH groups on its surface [70,71]. However, unlike bacterial cellulose, plant cellulose is impure due to the presence of lignin, hemicellulose and pectin. Apart from that, plant cellulose is slightly cytotoxic, less malleable and has small pore sizes due to less space between fibrils [72].

Nanocellulose can be prepared via mechanical or chemical methods. The most commonly used mechanical methods include high intensity ultrasonication, high-pressure homogenisation, micro-grinding and PFI milling [62]. However, these processes require high-energy consumption [73,74]. Chemical methods of nanocellulose preparation, such as acid hydrolysis, enzyme hydrolysis and (2,2,6,6-tetramethylpiperidin-1-yl) oxidanyl (TEMPO) oxidation, have also been rigorously employed. Yet, the drawbacks of the chemical methods are that they are time-consuming and yield low results [75]. Therefore, chemical and mechanical methods have been combined to overcome these issues, as well as reduce energy consumption. Acid hydrolysis is the most common type of this method used to date. The hydrolysis method reduces the sizes of nanofibres from microns to nanometres [76,77,78,79]. However, this method is not without drawbacks, such as the use of high concentrations of acid, which leads to acid waste and considerably adverse effects on the environment.

## 2. Preparation of Graphene Quantum Dots–Nanocellulose Composites

Over the past few years, GQDs–nanocellulose composites have been used in a variety of applications that have since generated a lot of interest in their synthesis and composite processing. Multiple studies have proven that GQDs–nanocellulose composites perform better than both GQDs and nanocellulose on their own. Different preparation techniques could impart different abilities to a composite as the preparation technique can affect the particle size, as well as the mechanical properties [80]. However, the major issues encountered when preparing nanocellulose composites include enhancing compatibility with hydrophobic polymers, uniform dispersion within the matrix and large-scale production [81]. Although melt-blending and in situ techniques can be employed during composite preparation, solution blending is the most common technique of GQDs–nanocellulose composite preparation. However, specific conditions, such as maintaining the temperature of the substrate, solution and solvent, are required during solution blending. Furthermore, material concentration and pH also affect the efficacy of the final composite.

**Figure 2:**
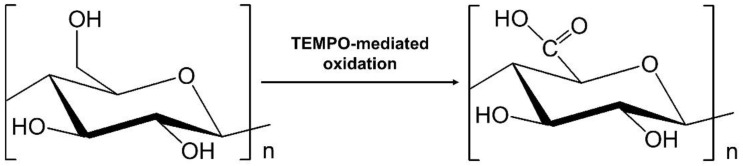
Oxidation of C6 hydroxyl group of cellulose in a TEMPO-system.

Solution blending facilitates molecular mixing, which ensures that a composite is soluble in the appropriate solution. According to Ruiz-Palomero, Benítez-Martínez et al., GQDs–nanocellulose hydrogel particles can be prepared by combining carboxylated nanocellulose into sulphur–nitrogen co-doped GQDs (S, N-GQDs) [30]. Another study found that immersing S, N-GQDs synthesized via a hydrothermal process after using thiourea as the S and N source and citric acid as the source of carbon into the nanocellulose improved the penetration of the excitation light in the hydrogel composite and maintained higher fluorescence energy [82]. Carboxylated nanocellulose can be prepared using (2,2,6,6-tetramethylpiperidine-1-oxyl) TEMPO-radical catalysed oxidation. TEMPO was found to selectively oxidise the C6 primary alcohol moiety that is present on the surface of the nanocellulose particles and formed carboxylated groups [83], as presented in Figure 2.

The regioselective conversion of the primary hydroxyl group to carboxylate weakens the adhesion between nanocellulose fibrils, as it prevents the formation of interfibrillar hydrogen bonds [84]. The TEMPO-mediated oxidation of the cellulose slurry starts to form when the NaOCl solution is added at room temperature and the pH is maintained at 10. The temperature is maintained at 25 °C, as the contents of carboxyl groups increases as the temperature increases up to only 25 °C and subsequently decreases at 35 °C. As such, optimum temperature is critical to form complete reactions, as increases in temperature decrease carboxyl group content, leading to de-polymerisation and losses in total yield. Meanwhile, lower temperatures may cause incomplete reactions to occur, since unreacted NaOCl forms and NaOH needs to be added to the solution to neutralise the generated carboxylic acid groups and maintain a pH of 10. The amount of NaOH added is gradually increased and remains constant after a certain time, once the limiting reactant, NaOCl, is fully consumed [82]. This technique provides a more uniform dispersion of nanocellulose in an aqueous phase. After conducting an inversion test on the composite hydrogels, Ruiz-Palomero, Benítez-Martínez et al. found that the hydrogels could be reformed several times as non-covalent interactions were involved [30].

The same researchers also reported a similar GQDs–nanocellulose hydrogel preparation procedure for sensing 2,4,5-trichlorophenol [31]. In short, the carboxylated nanocellulose was mixed with an aqueous solvent that contained S, N-GQDs, where the nanocellulose acted as a gelator. The sample was mixed using a vortex and sonication, then centrifuged for 0.5 min at 1300 rpm and heated for 20 s in a vial. The transparent hydrogel was observed following cooling to room temperature after the last centrifugation stage. An inversion test was used to assess gel formation. Due to the interacting surface hydroxyl and carboxyl groups, the carboxylated nanocellulose was found to be an effective gelator. This caused a significant self-association that resulted in nano-fibre entanglement due to hydrogen bonding. Therefore, nanocellulose is a suitable gel matrix for hosting GQDs. The study also found that 10 *wt*% of carboxylated nanocellulose provided the most stable hydrogel when mixed with the S, N-GQDs. This successful gel formation and stability confirmed the compatibility and suitability of the nanocellulose in hosting the GQDs and that it was potentially capable of detecting various analytes. This was due to its exceptional optical features which were accentuated by the network formation combined with the photoluminescence behaviour of the GQDs, which rendered them suitable for analytical purposes.

Apart from nanocellulose and GQDs concentration, the pH of the solvent containing GQDs and the doping characteristics of the GQDs play a significant role in the successful formation of a GQDs–nanocellulose hydrogel composite. The integrity of a composite structure can be weak in some cases due to the pH of the media or material concentration. For instance, Ruiz-Palomero, Soriano et al. [31] reported that only a solution of dialysed GQDs that had a neutral pH could support gel formation. Moreover, pure undoped GQDs produced weak gels, while N-doped GQDs did not form hydrogels even after purification. However, the S-GQDs produced low photoluminescence albeit gels. Doping GQDs with a combination of S and N heteroatoms (S, N-GQDs) produced the most stable gels with strong fluorescence features. On the other hand, the concentration of GQDs needed to be optimised as a very high concentration may de-stabilise the hydrogen bonding network via additional π–π stacking interactions. The study revealed that an 8 mg mL^−1^ concentration of S, N-GQDs and 10 *wt*% carboxylated nanocellulose produced the best and strongest hydrogel with the highest photoluminescent features.

Tetsuka et al. [85] was one of the earliest attempts at producing a GQDs–nanocellulose composite via solvent blending by producing a transparent clay film comprising of amino-functionalised GQDs (af-GQDs) and cellulose nanofibrils (CNF) as a colour converting material for blue light-emitting diode (LED) [85]. Treating the heavy oxidised graphene sheets (OGSs) with a mild amino-hydrothermal method produced uniform-sized af-GQDs with a tuneable photoluminescence. The amino groups were found to alter the electronic structures and shift the HOMO levels to a higher energy with a maximal photoluminescence at the long wavelength [85]. The af-GQDs–CNF composite was prepared by mixing the CNF suspension with aqueous solution containing af-GQDs. A clay suspension was prepared by using a high shear mixer to disperse clay in a solution containing 15 *wt*% of the af-GQDs–CNF. The mixture was then degassed and centrifuged to remove any flocculated clay impurities before it was poured on a glass mould. It was then heated at 60 °C overnight and detached from the glass mould to produce af-GQDs–CNF clay film. Unlike Ruiz-Palomero, Soriano et al. [31], Tetsuka et al. [85] introduced the incorporation of clay in the composite matrix via electrostatic interactions between the clay and af-GQDs–CNF to form a flexible and transparent film. The resultant film exhibited bright colourful photoluminescence and is a promising future light emitting diode application.

Khabibullin et al. [86] produced injectable fluorescent hydrogels composed of GQDs and cellulose nanocrystals (CNCs). Unlike Ruiz-Palomero, Soriano, et al. [31] and Tetsuka et al. [85], who used the hydrothermal method, Khabibullin et al. [86] employed the Hummers method to prepare the GQDs. The CNCs were functionalised with amino groups before being subjected to the composite hydrogel preparation. The hydrogel was prepared by dispersing the powdered GQDs in aqueous solution containing CNCs. The sample was mixed via vortex, then left to rest. The time of hydrogel formation varied depending on the concentration of GQDs and CNCs. The sample with 50 mg/mL of CNCs and 7 mg/mL of GQDs yielded a strong hydrogel formation (gelation within 30 min), while the sample with lower concentrations of CNCs and GQDs yielded a weak hydrogel formation (gelation within four hours) and the sample with <20 mg/mL CNCs and <5 mg/mL GQDs yielded no hydrogel formation. Similar to the neutral pH that facilitated hydrogel formation in Ruiz-Palomero, Soriano, et al.’s [31] study, the pH used for the preparation of hydrogel was maintained at seven. As the produced GQDs–CNCs composite possessed a shear thinning behaviour, it was a suitable injectable material for 3D printing with additional fluorescence features.

Alizadehgiashi et al. [8] also employed a solution mixing procedure for the preparation of GQDs–nanocellulose composites. Similar to Tetsuka et al. [85], Alizadehgiashi et al. [8] utilised amino-functionalised GQDs (af-GQDs) that had been prepared using the hydrothermal method, except that an aldehyde-modified CNCs was used for the composite hydrogel formation instead. The composite hydrogel was prepared by mixing various concentrations of aldehyde-functionalised CNCs (from 10 to 60 mg/mL) and af-GQDs (from 2.5 to 60 mg/mL) suspensions in various volumetric ratios, which may change the structure of the composite from lamellar to nanofibrillar, as well as enabling the controlling of the permeability of the hydrogel. A thick lamellar structure (large pores) was observed at a lower CNC concentration ratio, while a nanofibrillar structure (small pores) was observed at a high CNC concentration ratio (low af-GQDs content). The transition of the structure can be attributed to the number of crosslinking points available (higher CNC content imparts fewer crosslinking points), which, in turn, determines the thickness of the wall and size of the pores. In terms of permeability, the hydrogel composite with lower CNC content (lamellar structure) had higher permeability than the composite with higher CNC content (nanofibrillar structure) due to the larger pore size [87].

On the other hand, Rosddi et al. [88] produced a GQDs–nanocellulose composite by dissolving carboxylated GQDs in an aqueous solution containing cationically modified CNCs. A thin film of the composite was then formed using the spin coating technique and the composite showed potential in analytical applications. The same researchers later reported the modification of surface plasmon resonance gold film with carboxylated GQDs–CNCs to enhance the detection sensitivity of glucose [89]. Similar to Ruiz-Palomero, Benítez-Martínez, et al. [30] and Ruiz-Palomero, Soriano, et al. [31], Mahmoud et al. [90] prepared GQDs–nanocellulose using S, N-GQDs, except that the nanocellulose used was not modified with the carboxyl group and no hydrogel formation was reported. The GQDs–nanocellulose was prepared by mixing of equal concentrations of S, N-GQDs and nanocellulose (3 mg/mL each). The composite was then sonicated and drop-casted onto glassy carbon electrode (GCE) to fabricate a modified GCE sensor. More recently, Xiong et al. [91] prepared a flexible GQDs–nanocellulose film via a combination of electrolysis and liquid dispersion for sensor and supercapacitor applications.

**Figure 3:**
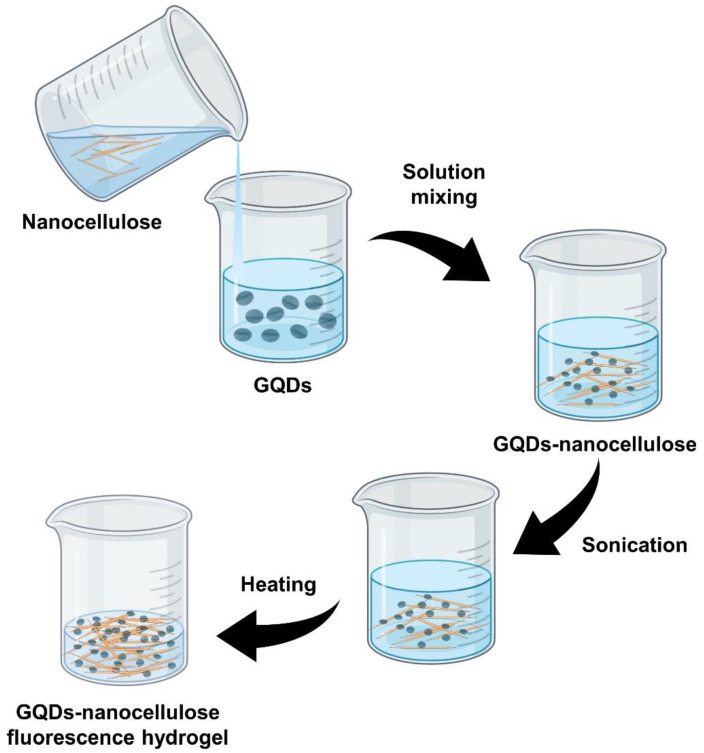
Preparation of GQDs–nanocellulose fluorescence hydrogel via solution mixing.

The average particle size of GQDs–nanocellulose composites can be controlled by altering the reaction conditions. The reactive chemical groups and its distinctive morphology make nanocellulose a good biological template for GQDs–nanocellulose synthesis. Solution mixing is the most common method of preparing nanocomposite films by far. This method is suitable for obtaining good dispersion of nanocellulose with a polymer solution due to the good dispersion of these nanoparticles in water. Figure 3 presents a schematic illustration of GQDs–nanocellulose hydrogel formation.

## 3. Marriage Chemistry of GQDs and Nanocellulose

As GQDs can significantly affect the properties of a composite, determining the GQD content threshold of a nanocomposite depends on its specific application, as different concentrations are required for different applications. The GQD, a functional derivative of graphene, consists of many oxygen-containing functional groups, such as ether, hydroxyl, carbonyl and carboxyl, on the edges and basal planes [92,93]. Despite the smaller size of GQDs, their edge sites are more reactive than the carbon matrix [94]. Furthermore, the presence of functional groups not only facilitates the easy blending of GQDs into a polymer matrix [95,96] but also improves its electrical, mechanical and dielectric properties. The equatorial direction of the glucopyranose ring present in the nanocellulose exhibited a hydrophilic nature, as all three hydroxyl groups were placed at an equatorial position on this ring, while the hydrogen atoms in the carbon and hydrogen bonds were located at the axial positions of the rings, which is responsible for the hydrophobic nature of the axial direction of the glucopyranose ring [86].

**Figure 4:**
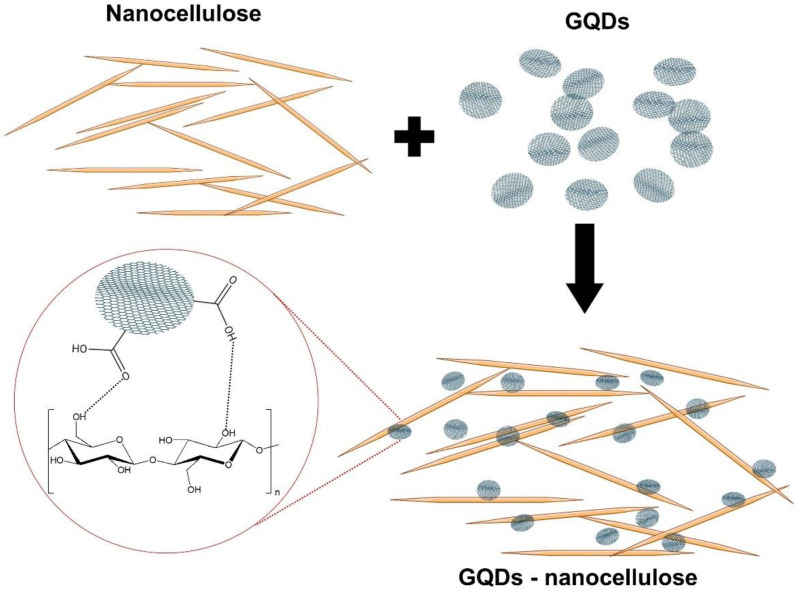
A schematic illustrating the interaction and formation of the hydrogen bonding between the carboxyl groups of graphene quantum dots (GQDs) and the hydroxyl groups of nanocellulose.

As illustrated in Figure 4, the attractive forces from the hydrogen bonds between the hydroxyl groups on the surface of nanocellulose particles and the oxygen-rich carboxyl groups present on the GQD edges produce a strong and stable GQDs–nanocellulose composite. Hydrophobic interactions are noted between the basal planes of GQDs and hydrophobic faces of the nanocellulose [86]. Although repulsive forces may occur between the negatively charged sulphate half-ester groups present on the surface of the nanocellulose surface and the carboxylic groups present on GQDs due to electrostatic interactions, the hydrogen bonds and the hydrophobic forces can overcome the repulsive forces leading to the crosslinking of the GQDs and the nanocellulose. The functional groups containing the oxygen atom in GQDs and the aromatic sp^2^ domains can generate an interfacial bonding in the GQDs and nanocellulose composites [31]. The network noted in nanocellulose composites was seen to alter the structural region and form amorphous and crystalline regions, thereby increasing the mechanical strength of the composites [97].

Therefore, the addition of GQDs improves the tensile strength, toughness and stiffness of nano-cellulosic composites due to the hydrogen bonds between the functional groups of both GQDs and nanocellulose. The intralayer of the hydrogen bonds present in the nanocellulose is constant, while the hydrogen bonding in the nanocellulose interlayers can be significantly improved. Additionally, the interaction between GQDs and nanocellulose induces a locally stable energy state at the interfacial plane of nanocellulose. The marriage chemistry of GQDs-nanocellulose composites is summarised in Table 1.

The concentration of GQDs and nanocellulose may affect the interactions within a composite. For instance, a higher concentration of GQDs may induce the formation of π–π stacking between GQDs, thus affecting the hydrogen bonding network of the nanocellulose and hydrogel formation. Although such an interaction (π–π stacking) and graphitic aggregation are accounted for and may be accentuated, the surface modification and functionalisation of a nanomaterial also affect the inter-nanomaterial interactions. For example, the carboxyl and hydroxyl groups of carboxylated nanocellulose interact with the oxygen-containing groups of GQDs and these interactions may facilitate significant improvements and provide a synergistic enhancement of the composite’s properties. Therefore, this excellent combination of GQDs and nanocellulose appears to be an outstanding strategy because it results in the improvement of the properties of each individual component, including the mechanical strength of the composite or hydrogel, as well as the enhancement of optical or fluorescent features [31,85]. GQDs–nanocellulose composites are promising for sensing, printing materials and analytical applications by virtue of their unique optical, structural and mechanical properties. The synergistic manner and improvement of the fluorescent properties of a composite can be indicative of the feasible interaction and marriage chemistry between the superficial groups of GQDs and the oxygen-containing groups of nanocellulose, thus reducing π–π stacking-induced GQD aggregation.

Although cellulose is usually described as a hydrophilic material due to the presence of hydroxyl groups, it is noteworthy that the significant amphiphilicity of cellulose is due to the hydrophobic effects of C–H grouped in the glucopyranose unit backbone of the cellulose. This cellulose behaviour, as dictated by the hydrophobic effect, was conceptualised by Lindman et al. [98] and subsequently prompted a unique and intriguing debate among cellulose experts [99]. The hydrophobicity of cellulose is also corroborated by Yamane et al. [100], based on the anisotropy of cellulose’s structure. Despite its hydrophilicity, cellulose has been found to interact strongly with non-polar (hydrophobic) organic solvents, such as toluene, dichloromethane and hexane [100]. An investigation of its structural anisotropy proposed that the hydrophobic behaviour of cellulose stems from the C–H bonds of the glucopyranose rings located on the axial position of the rings. On the other hand, it is well known that graphene materials, including GQDs, have hydrophobic properties. Owing to the hydrophobicity of graphene and amphiphilicity of cellulose, the marriage chemistry and interaction between these two materials can be elucidated. Moreover, Alqus et al.’s [101] theoretical modelling and molecular dynamic simulation investigated the interactions between cellulose and graphene. The study reported stable complex formation of graphene-cellulose through a hydrophobic interface which was primarily formed by CH–π interactions. Therefore, this further emphasises that the amphiphilic nature of cellulose plays an important role in favoured interactions when fabricating a GQD–cellulose composite.

## 4. Applications of GQDs–Nanocellulose Composite

**Figure 5:**
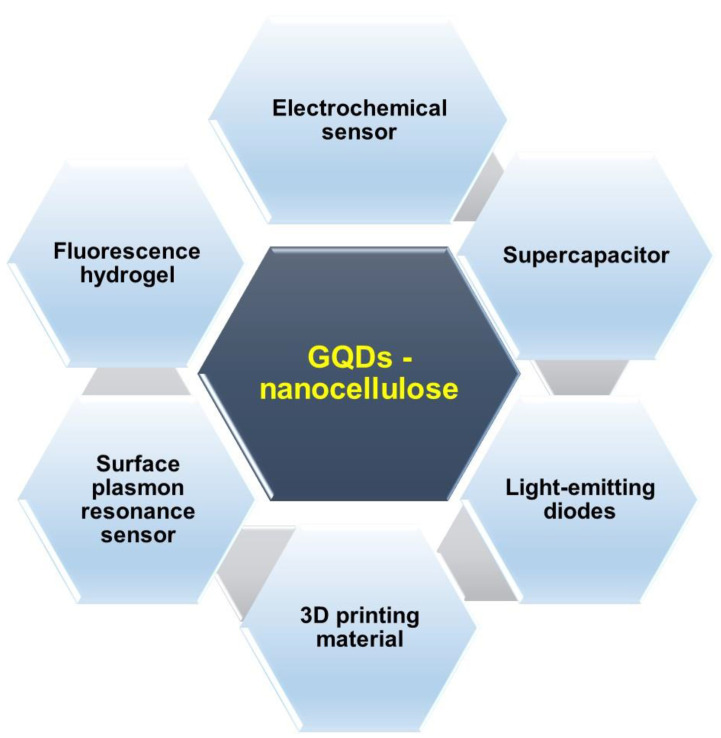
The applications of GQDs–nanocellulose composites.

Nanocellulose and GQDs have garnered a lot of research interest in different fields, such as energy storage devices, electronics, photovoltaic devices and biosensors [102,103,104]. The various functional groups present on the surface of GQDs, such as -OH, -COOH and -NH_2_, act as active coordination sites for the transition metal ions. Nanocellulose is biocompatible, environmentally friendly, flexible and thermally stable [105,106,107]. Nanocellulose can improve mechanically flexible materials, as they are receptive to light, heat, chemicals and magnetic fields which can connect stimulus responses and allow the composites to work optimally [108]. These intriguing properties of GQDs–nanocellulose composites have encouraged researchers to deeply explore and unlock their potential for use in a multitude of applications, including sensors, light-emitting diodes, 3D printing materials and supercapacitors, as summarised in Figure 5.

### 4.1. Sensors

As seen in Table 2, GQDs–nanocellulose composites can be used to produce sensors with which to detect various analytes. Multiple types of sensors that were developed from GQDs–nanocellulose, such as drug sensors, humidity sensors, laccase monitoring sensors, glucose sensors and metal ion sensors, were investigated. The functional capabilities of each sensor improved when GQDs were added into the nanocellulose matrix. These sensors are materials or electronic devices that convert one form of energy to another [102]. The conditions required to develop the best sensor include stability and sensitivity, so that they can detect trace quantities of molecules in various applications. Apart from that, the sensors also need to provide accurate results with rapid performance [108].

#### 4.1.1. Modified Electrochemical Sensor for Drug Detection

An electrochemical sensor functions when gases react or generate electrical signals according to a concentration of gas. These sensors consist of a counter electrode and electrodes separated by an electrolyte layer [109]. The gas concentration is proportional to the electrical signal that is generated. The mechanism of action of an electrochemical sensor is that the gas which interacts with the sensor passes through the capillary opening and diffuses through the hydrophobic barrier before it reaches the surface of the electrode [110]. This ensures a proper gas flow, that can react with the sensing electrode. An appropriate amount of electrical signal is generated in this procedure, without causing any leakage [111]. The larger number of active sites, higher conductivity for charge transfer to the electrodes and the stable mechanical strength of GQDs–nanocellulose composites help electrochemical sensing, as the structure of the electrode facilitates high electrical conductivity and rapid electron transfer at the surface of the electrodes. Electrochemical methods have garnered considerable interest in recent times due to their high sensitivity, simplicity and rapid analysis. Mahmoud et al. [90] recently used a S, N-GQDs–nanocellulose composite to modify electrochemical sensors for drug analysis. The nanomaterial was drop-casted onto the surface of a bare electrode and dried prior to analysis. It was revealed that the modified electrode sensor provided good electro-catalytic performance for drug detection. Furthermore, the active surface area increased two-fold, in comparison to the bare electrode. The modified electrode also showed good reproducibility and repeatability with relative standard deviations of less than 2.4% and 2.1%, respectively. The electrode maintained its original performance of 95% within 28 days of storage, indicating good stability. This can be explained by the intermolecular interactions of various functional groups, unique structure and high active surface area due to the addition of S, N-GQDs and nanocellulose, which enhanced the analytical performance of the electrode in a synergistic manner. 

An earlier study developed an electrochemical sensor using only GQDs and found poor electrical conductivity. The performance of the sensor was improved by adding heteroatoms to the core of the GQDs. The oxygen-rich functional groups of the GQDs could improve the properties of the electrochemical sensor, wherein the sensors showed good quantum confinement, edge effect and a larger surface area [90]. Furthermore, the addition of nanocellulose particles to the composites improved the sensitivity of the electrochemical sensors in detecting many analytes. The nanocellulose particles contained many OH groups which were modified to ensure the selective binding of the analytes. The GQDs–nanocellulose composites improved ionic conductivity as they increased ion transportation by introducing many ion conduction pathways. The addition of heteroatoms, such as nitrogen, sulphur, boron and phosphorous, to the composite helped form strong bonds due to hydrogen bonding or π–π stacking. Therefore, GQDs–nanocellulose composites facilitate the adsorption and electrochemical oxidation of drugs onto the surfaces of modified electrodes due to the presence of π–π stacking or hydrogen bonds.

#### 4.1.2. Hydrogel Sensor for Enzyme Detection

Combining GQDs and nanocellulose to develop fluorescent hydrogels has quickly become a new strategy of detecting laccase enzymes over the last few years [30]. The unique optical features provided by GQDs, as well as the three-dimensional framework provided by nanocellulose, enable significant detection of laccase through fluorescent quenching and ideal enzyme immobilisation, respectively. In short, the fluorescent intensity decreased as the concentration of laccase enzymes increased. The hydrogels also possessed remarkably better signal stability than non-nanocellulose framework platforms due to the enzyme immobilisation behaviour imparted by the nanocellulose framework platform. These hydrogels are described as a heterogeneous mixture of multiple phases. The dispersed phase in the hydrogel is water, whereas the solid 3D network forms a solid phase. The hydrogel consists of a 3D polymeric network which is filled with water and displays a gel-like behaviour due to the hydrogen bonding between the nanofibrils in the aqueous medium. Water retention occurs due to the formation of hydrogen bonds between the hydrophilic groups in these composites. As such, hydration can cause the deformation of the polymeric chains when compensating the stresses within this structure [112]. Fluorescent hydrogels that have been developed using GQDs–nanocellulose composites are reversible and can easily convert to form a fluid when heated or stirred at a higher shear rate. This process can be repeated up to 10 times without issues owing to the presence of non-covalent interactions between the molecules [30].

Hydrogel fluorescent sensors are cost-effective, simple and eco-friendly. They can also detect and stabilise laccase molecules, as well as storing or recycling the enzymes. It is also serves as a stabilising matrix for trapping enzyme molecules from the complex matrix and reusing it after being stored. This helps protect the enzyme molecules from extreme environmental conditions. Therefore, the enzyme molecules can be stored in a nano-cellulosic hydrogel and recovered for use at a later stage [30]. Laccase detection was attributed to the quenching of fluorescent molecules in the GQDs–nanocellulose hydrogels. This was caused by the interactions between the laccase enzymes and the graphitic layers which are stabilised by the nanocellulose. Another advantage of using a fluorescent GQDs–nanocellulose hydrogel is that it follows a simple mechanism, as the enzymatic activity does not have to be measured. Furthermore, it is eco-friendly and can be easily prepared using biocompatible nanoparticles which are self-organised due to electrostatic interactions [113].

#### 4.1.3. Hydrogel Sensor for Pesticide Detection

The use of GQDs–nanocellulose composites for developing a hydrogel sensor to detect 2,4,5-trichlorophenol (TCP) has also been investigated [31]. An organic compound belonging to the chlorophenols family, TCP is carcinogenic to humans [114]. As such, it is commonly used in fungicides and herbicides or as an intermediate in the production of other pesticides [115]. As TCP has been detected in soil, groundwater and drinking water, it poses a health and environmental risk. Therefore, it is imperative to detect and remove these hazardous compounds from the environment. Consequently, the development of a GQDs–nanocellulose hydrogel as sensing platform is as strategic endeavour that is convenient, rapid and simple with a selective sensing ability for TCP determination. Such fluorometric hydrogel sensor provided remarkable performance, including high sensitivity, good repeatability and reproducibility, indicating the immense potential of composites as sensing analytical tools. Although this hydrogel, in particular, is selective towards TCP, its potential to detect various other types of pesticides should be further explored. By virtue of its versatility, unique properties and further surface function modification, GQDs–nanocellulose may serve as a promising sensing candidate for pesticide detection.

#### 4.1.4. Hydrogel Sensor for Metal Ion Detection

In recent years, Alizadehgiashi et al. [8] produced a nano-colloidal heavy metal ion scavenger hydrogel composed of GQDs and CNCs. This hydrogel possessed more active surface sites as well as a larger surface area and metal ion sensing capacity. The presence of carboxylic acid groups on the edges of the GQDs allowed them to serve as metal ion scavengers, while nanocellulose or CNCs provided the hydrogel with immobilisation capabilities that facilitate easy water separation. Therefore, the GQD and CNC content determined the number of active sites and the pore size of the hydrogel composite, respectively.

#### 4.1.5. Humidity Sensor

Earlier studies describe two types of humidity sensors, capacitive and resistive [116,117]. Humidity sensors are used in the window defogger system of the automobile industry, in respiratory equipment and for medicine processing in the medical field, as well as controlling greenhouse air and monitoring soil conditions in the agricultural field. Existing studies have investigated the synergistic benefits of graphene oxide–nanocellulose composite films and their application while designing flexible, cheap and renewable humidity sensors.

Kafy et al. [95] found that graphene oxide–CNCs composite films had higher sensitivity than CNC films while developing a humidity sensor. In general, the inclusion of the graphene oxide into the CNC matrix increased the number of hydrophilic functional groups, such as carboxyl and hydroxyl. It was observed that the sensing capability of CNCs was dependent on the hydrophilic functional groups that attracted water molecules and improved the capacitance. Although the efficacy of using a GQDs–nanocellulose composite as a humidity sensor has yet to be investigated, the presence of carboxyl and hydroxyl groups in GQDs, both of which supported the chemical surface membrane of CNCs, may enable it to outperform the GO–nanocellulose humidity sensor. Furthermore, GQDs–nanocellulose composite films, which have a higher rigidity and mechanical strength, could be used for long-lasting applications.

#### 4.1.6. Surface Plasmon Resonance Sensor for Glucose Detection

Rosddi et al. [88] recently fabricated a thin GQDs–nanocellulose composite film using a combination of carboxylated GQDs and cationically functionalised nanocellulose. The same researchers later used GQDs–nanocellulose composites to modify a surface plasmon resonance gold film sensor to enhance the detection sensitivity to glucose [89]. Their results indicated that the thin GQDs–nanocellulose film had better sensitivity and glucose binding affinity than an unmodified sensor, which can detect various concentrations of glucose with the lowest detection of 5 nM.

### 4.2. Light Emitting Diode

One of the earliest studies on GQDs–nanocellulose composites showed its significant potential when incorporated into a flexible film as a colour-converting material in light-emitting diode (LED) [85]. The film was prepared by mixing amino-functionalised GQDs and cellulose nanofibres into the clay matrix. The resultant film exhibited excellent physical stability, as well as bright photoluminescence, and emitted a white light when casted onto a blue LED. Therefore, the incorporation of GQDs–nanocellulose into a composite film is promising for the future of light emitting devices, such as bio-imaging, photovoltaics and colour-converting material.

### 4.3. Three-Dimensional Printing Material

GQDs–nanocellulose has also been investigated for its potential as an injectable 3D printing material [86] by controlling its gelling properties and shear-thinning behaviour. The performance of the injectable material was investigated using a 3D printer. The composite was able to form a pre-designed pattern and retain its thread-like shape. Furthermore, the extruded thread exhibited photoluminescent properties due to the fluorescence characteristics of GQDs. The birefringent properties observed on the hydrogel thread under polarised optical microscopy stemmed from the shear-induced alignment of CNCs within the composite. In light of all these advantages, GQDs–nanocellulose is a useful injectable material and intriguing candidate for a wide range of applications, including tissue engineering, bio-imaging, drug delivery, biomedical and 3D printing.

### 4.4. Supercapacitor

Xiong et al. recently combined electrolysis and liquid dispersion to produce a composite film comprising of cellulose nanofibres and GQDs for use as a supercapacitor [91]. The produced hybrid film had remarkable electrochemical storage performance and mechanical properties. It also exhibited a specific capacitance of 118 mF cm^−2^ even at an ultrahigh scan rate of 1000 mV s^−1^ and a high capacitance retention of more than 93% after 5000 cycles at various current densities. Moreover, the supercapacitor constructed based on the GQDs–nanocellulose presented high energy densities and power indicating exceptional performance rate and cycle stability. Therefore, combining GQDs and nanocellulose not only overcame the drawbacks of individual materials, such as low efficiency and poor conductivity, but enhanced performance in a synergistic manner. This indicates that the scientific community should further investigate the suitability of GQDs–nanocellulose for fundamental studies as well as advanced applications.

## 5. Conclusions

Owing to unique characteristics, such as inherent luminescence, biocompatibility, high surface area, high adsorption, good surface functionality, significant strides have been made in the preparation and application of GQDs–nanocellulose over the past several years. Studies have shown that the hybridisation of these novel materials not only improves existing applications but also provides additional advantages, as well as further improvement of desirable features, all of which are unattainable if GQDs and nanocellulose are used individually. Therefore, this advantageous composite material warrants remarkable applications. This review provided a brief overview of this evolving body of research which will unlock windows of opportunities for future research and multifunctional applications. GQDs–nanocellulose can be produced via rapid and simple solution blending or drop-casting onto a selected matrix depending on the targeted application. Furthermore, nanomaterial concentration, composition ratio, pH of the media, heteroatoms doping and surface functionalisation determine the properties, marriage chemistry and final GQDs–nanocellulose composites. The remarkable and synergistic properties of GQDs–nanocellulose certainly unlock their potential for use in a multitude of applications, including sensors, light-emitting diodes, injectable shear-thinning materials and supercapacitors. Although the development of GQDs–nanocellulose composites is limited and still at its infancy, this hybrid material is anticipated to be commercially accessible and more practical in the future. This review disclosed a range of opportunities to apply nanomaterials derived from naturally occurring cellulose polymers or renewable biomass resources and assisted in more innovative nanomaterial production methods and developments in the future.

## Figures and Tables

**Table 1:** A summary of marriage chemistry of GQDs–nanocellulose composites.

Composite	Marriage Chemistry	Ref.
Amino-functionalised GQDs + aldehyde-modified CNCs	Chemical crosslinking due to hydrogen bonding between amino groups of the amino-functionalised GQDs and hydroxyl groups of aldehyde-modified CNCs. Ionic interactions between negatively charged sulphate half-ester groups of CNCs and protonated amines of GQDs. Hydrophobic interactions between basal planes of GQDs and hydrophobic backbone of CNCs.	[8]
S, N-GQDs + carboxylated nanocellulose	Electrostatic interactions between superficial groups of GQDs and oxygen-containing nanocellulose.	[30]
S, N-GQD + carboxylated nanocellulose	Hydrogen bonding interactions between carboxyl and hydroxyl groups of nanocellulose and oxygen-containing groups of GQDs. Hydrogen bonding network de-stabilised with higher GQDs content due π–π stacking-induced GQD aggregation.	[31]
GQDs + amino-modified CNCs	Hydrophobic interactions between basal plane of GQDs and C–H grouped located on axial positions of glucopyranose ring of CNCs. Hydrogen bonding interactions between amino or hydroxyl groups of CNCs and oxygen-rich groups on GQD edges.	[86]
Carboxylated GQDs + cationically modified CNCs	Hydrogen bonding interactions between oxygen-containing groups of GQDs and hydroxyl and oxygen atoms in CNCs.	[88]
Carboxylated GQDs + cationically modified CNCs	Hydrogen bonding interaction between carboxylated GQDs, and hydroxyl groups and oxygen atoms in CNCs	[89]
S, N-GQDs + nanocellulose	Hydrogen bonding formation between polar groups of S, N-GQDs and nanocellulose. π–π stacking between GQDs	[90]

**Table 2:** A summary of various GQDs–nanocellulose composite-based platforms used for sensing applications.

Sensor	Prior to GQDs–Nanocellulose Composite Formation	Post GQDs–Nanocellulose Composite Addition
Drug	Electrochemical sensor with poor electro-catalytic performance and low active surface area for drug detection.	Good conductivity, increased active surface area and good reproducibility, repeatability and stability for drug detection.
Metal ions	Low detection limit and unable to measure many metal ions.	Large surface area and higher number of active sites on the membrane increased the efficiency of scavenging metal ions from samples.
Laccase	Based on catalytic activity which is dependent on environmental parameters and the presence of inhibitors and inducers.	Based on fluorescence response of hydrogels containing GQDs acting as luminophore towards laccase.
Humidity	Less sensitive and produce less accurate result.	Sensitivity is expected to increase due to the abundance of hydrophilic functional groups in both GQDs and nanocellulose.
Glucose	Low sensitivity and binding affinity.	More sensitive and higher binding affinity constant.

## Data Availability

Data supporting reported results can be found in publicly archived datasets such as ScienceDirect and Scopus.
